# Person-to-Person Transmission of Andes Virus in Hantavirus Pulmonary Syndrome, Argentina, 2014

**DOI:** 10.3201/eid2604.190799

**Published:** 2020-04

**Authors:** Daniel O. Alonso, Unai Pérez-Sautu, Carla M. Bellomo, Karla Prieto, Ayelén Iglesias, Rocío Coelho, Natalia Periolo, Isabel Domenech, Gabriel Talmon, Romina Hansen, Gustavo Palacios, Valeria P. Martinez

**Affiliations:** Instituto Nacional de Enfermedades Infecciosas "Dr. C. Malbrán," Buenos Aires, Argentina (D.O. Alonso, C.M. Bellomo, A. Iglesias, R. Coelho, N. Periolo, V.P. Martinez);; United States Army Medical Research Institute of Infectious Diseases, Frederick, Maryland, USA (U. Pérez-Sautu, K. Prieto, G. Palacios);; University of Nebraska College of Public Health, Omaha, Nebraska, USA (K. Prieto);; Hospital Zonal de Bariloche, Bariloche, Argentina (I. Domenech);; Ministerio de Salud de la Provincia de Río Negro, Rio Negro, Argentina (G. Talmon);; Hospital de Área El Bolsón, Rio Negro (R. Hansen)

**Keywords:** Andes virus, hantavirus, viruses, hantavirus pulmonary syndrome, person-to-person transmission, next-generation sequencing, Argentina

## Abstract

Andes virus is unique among hantaviruses because it can be transmitted from person to person. This mechanism was previously supported by epidemiologic data and genetic evidence based only on partial sequences. We used full-length virus sequencing to confirm person-to-person transmission of this virus in a cluster of 3 cases in Argentina in 2014.

Pathogenic hantaviruses are members of the family *Hantaviridae* and genus *Orthohantavirus*. These viruses are responsible for hantavirus pulmonary syndrome (HPS) in the Americas. In Argentina, HPS was first described in 1995 during an outbreak in the Andean region of Patagonia, leading to the characterization of Andes virus (ANDV) (*1*). Since then, >1,200 cases have been confirmed in Argentina (*2*,*3*).

Hantaviruses are enveloped, single-stranded, RNA viruses with tripartite negative sense genomes. The small (S, 1.8–2.1 kb) segment encodes a nucleocapsid protein, the medium (M, 3.6–3.8 kb) a glycoprotein precursor, and the large (L, 6.5–6.7 kb) an RNA-dependent RNA polymerase (*4*).

Humans usually become infected with hantaviruses through inhalation of aerosolized excreta produced by infected rodents. ANDV is the unique hantavirus capable of being transmitted from person-to-person (*5*–*7*). Infection by this route takes place during the early prodromal phase, and the incubation period ranges from 9 to 40 days (*8*).

In previous outbreaks, genetic analysis was performed on partial sequences of ANDV, which represented ≈10% of the genome. Thus, the aim of this study was to analyze a cluster of 3 case-patients for whom epidemiologic data were available and compare complete viral genome sequences to assess person-to-person transmission or co-exposure to the same rodent population.

## The Study

The occurrence of 3 clustered cases during 2014 led us to suspect person-to-person transmission. The cases were reported during a 43-day period in El Bolsón, Rio Negro. The 3 case-patients had severe disease; 2 of these case-patients (P1 and P2) died (Table 1). P1 and P2, who were twin brothers, had symptoms develop 2 weeks apart, and each sought care at a primary healthcare center (Hospital Area El Bolsón, Rio Negro). P3 was a nurse who attended P2 during this initial hospitalization. After the beginning of the cardiopulmonary phase, each patient was transferred to a high-complexity hospital in Bariloche (Hospital Zonal Bariloche). P1 died 5 days and P2 7 days after symptom onset; P3 survived.

**Table 1:** Epidemiologic characteristics of 5 patients with hantavirus pulmonary syndrome, Argentina, 2014*

Patient	Age, y/sex	Date of symptom onset	Date of death	Relationship	Place of residence or work	Risk activity
P1	71/M	2014 May 10	2014 May 15	Twin brother of P2	Los Repollos forest reserve, Río Negro Province	Collecting firewood
P2	71/M	2014 May 25	2014 Jun 1	Twin brother of P1	Los Repollos forest reserve, rural area, Río Negro Province	Collecting firewood, contact with P1
P3	53/F	2014 Jun 16	Survived	Nurse of P2	El Bolsón, semiurban area, no evidence of rodent exposure in her home, nurse at HAEB	Assisted P2 at HAEB on May 27 and 28, before his transfer to HZB
NRC1	41/M	2014 Apr 30	2014 May 5	None	El Blanco, rural area, Cholila, Chubut Province	Daily contact with rodents in poor habitat conditions
NRC2	36/F	2017 May 11	Survived	None	Epuyen, rural town Chubut Province, high school teacher	Camping activities in Los Alerces National Park

*HAEB, Hospital Area El Bolsón (primary healthcare center); HZB, Hospital Zonal Bariloche (high-complexity hospital).

For our investigation, we included 2 unrelated HPS case-patients (NCR1 and NCR2) from the same area for comparison. We confirmed HPS in all 5 patients by using laboratory detection of ANDV-specific IgM and reverse transcription quantitative PCR, as described (*3*). All patients, except P2, had ANDV-specific IgG.

For whole-genome sequencing, we extracted RNA from peripheral blood of patients and prepared sequencing libraries by using target-enrichment technology and ANDV-specific probes. As expected, we identified the South variant of ANDV in the 5 case-patients. Comparative analysis showed 100% nucleotide identity in the whole genome between the samples from patients P2 and P3 (P2/P3 genome). Patient P1 had 100% nucleotide identity in the complete S and M segments with P2/P3 but had 2 nt changes in the L segment (99.95% nucleotide identity) (Table 2); both differences were silent mutations.

**Table 2:** Comparison of Andes virus south variant nucleotide and deduced amino acid sequences from patients with hantavirus pulmonary syndrome, southwestern Argentina*

Virus	Virus and genome segment																						
	P2/P3				P1				NRC1				NRC2				ANDV-South AH1 strain				ANDV-South Chile-9717869		
	S	M	L		S	M	L		S	M	L		S	M	L		S	M	L		S	M	L
P2/P3					100.0	100.0	99.96		95.4	94.5	94.7		98.6	98.7	98.7		96.4	95.4	NA		93.2	94.0	93.6
P1	100.0	100.0	100.0						95.4	94.5	94.7		98.6	98.7	98.7		96.4	95.4	NA		93.2	94.0	93.6
NRC1	99.5	98.95	98.7		99.5	98.95	98.7						95.1	94.5	94.5		95.5	94.7	NA		92.7	95.4	94.4
NRC2	100.0	99.6	99.5		100.0	99.6	99.5		99.5	98.6	98.6						96.2	95.2	NA		93.1	94.2	94.1
ANDV-South AH1 strain	100.0	98.4	NA		100.0	98.4	NA		99.5	98.8	NA		100.0	98.1	NA						93.3	94.5	NA
ANDV-South Chile-9717869	100.0	98.3	98.5		100.0	98.3	98.5		99.5	98.6	98.8		100.0	98.1	98.4		100.0	98.3	NA				

*Values above the spaces indicate percent nucleotide sequence identity, and values below the spaces indicate percent amino acid sequence identity. ANDV, Andes virus; L, large; M, medium; NA, not available; S, small. ANDV-South AH1, GenBank accession nos.: AF004660 (S); AF324901 (M); L: not available; ANDV-South Chile-9717869, GenBank accession nos.: AF291702 (S); AF291703 (M); AF291704 (L); ANDV-South P1, GenBank accession nos.: MN850083 (S), MN850088 (M), MN850093 (L); ANDV-South P2: GenBank accession nos.: MN850084 (S), MN850089 (M), MN850094 (L); ANDV-South P3, GenBank accession nos.: MN850085 (S), MN850090 (M), MN850095 (L); ANDV-South NRC1, GenBank accession nos.: MN850086 (S), MN850091 (M), MN850096 (L); ANDV-South NRC2, GenBank accession nos.: MN850087 (S), MN850092 (M), MN850097 (L).

On the basis of accurate epidemiologic findings, the only source of infection for P3 was her contact with P2 at Hospital Area El Bolsón. Despite the nucleotide differences between their isolates, co-exposure of P1 and P2 should not be discarded as a source of infection because these persons lived in the same house where they shared the same room and bed. However, even if one considers that these nucleotide changes were 2 silent mutations in the whole viral genome, person-to-person transmission is still the most probable way of infection for P2. A previous study reported a high degree of sequence diversity for the L segment of Puumala virus (*9*), which is consistent with our results.

**Figure 1:**
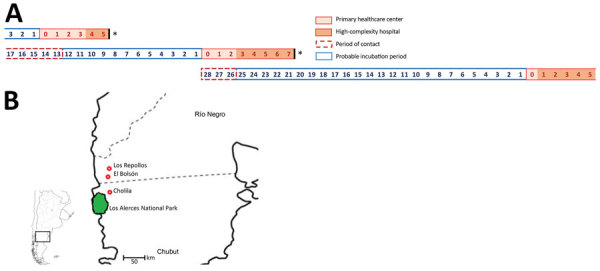
Temporal and geographic location of 2 cases of hantavirus pulmonary syndrome, southwestern Argentina, 2014. A) Timeline showing contact events, incubation periods, and period of illness for the 3 cases. Asterisks (*) indicate case-patients who died. B) Geographic location of patient residence or sites of exposure. Inset map shows study area in Argentina.

**Figure 2:**
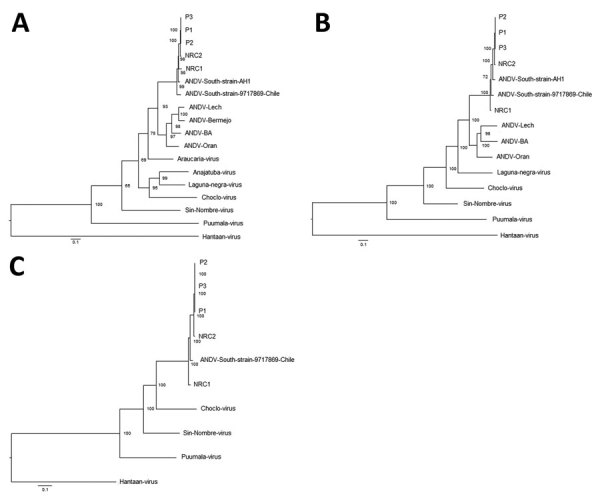
Phylogenetic analysis of hantaviruses based on complete genome of Andes virus (ANDV) isolated from case-patients in Argentina, 2014, and other orthohantaviruses characterized previously. A) Small (S) segment; B) medium (M) segment; C) large (L) segment. We used MrBayes version 3.2.7 (https://nbisweden.github.io/MrBayes) to reconstruct Bayesian maximum clade credibility trees. Numbers along branches are bootstrap values. Bootstrap support was based on 1,000 maximum-likelihood replicates. Scale bars indicate nucleotide substitutions per site. GenBank accession nos.: ANDV-South P1, S: MN850083, M: MN850088, L: MN850093; ANDV-South P2, S: MN850084, M: MN850089, L: MN850094; ANDV-South P3, S: MN850085, M: MN850090, L: MN850095; ANDV-South NRC1, S: MN850086, M: MN850091, L: MN850096; ANDV-South NRC2, S: MN850087, M: MN850092, L: MN850097; ANDV-Orán, S: AF325966, M: AF028024; Laguna Negra virus, S: NC038505, M: NC038506; ANDV-South, S: AF004660, M: AF324901; ANDV-South strain 9717869 Chile, S: AF291702, M: AF291703, L: AF291704; ANDV-Lech, S: AF482714, M: AF028022; ANDV-Bermejo, S: AF482713; Araucaria virus, S: AY740633; Anajatuba virus, S: JX443690; Choclo virus, S: KT983771, M: KT983772, L: EF397003; Sin Nombre virus, S: NC_005216, M: NC_005215, L: NC_005217; Puumala virus, S: NC_005224, M: NC_005223, L: NC_005225; Hantaan virus, S: JQ083395, M: JQ083394, L: JQ083393**.**

For further comparison, we obtained the complete sequences of 4 ANDV genomes circulating during a short period in the same area where this virus was first described (*1*). Nucleotide identity among strains from Argentina for the S and M segments ranged from 94.5% to 98.7%; for the L segment, it ranged from 93.6% to 98.7%. These comparisons included reference sequences for a strain from Chile. We observed a higher genetic identity between virus strains from patients P1, P2, P3, and NRC2. However, the site from which virus from NRC1 was isolated was closer to the sites of isolation of viruses from P1, P2, and P3 than to the site of isolation for virus from NRC2 (Figure 1). This finding confirmed the presence of different subtypes of the ANDV South variant co-circulating in nearby areas. Phylogenetic analysis showed that viruses from case-patients P1, P2, and P3 clustered together; NRC2 had the highest identity values for the 3 genomic segments (Figure 2).

The open reading frames encoding the nucleoprotein, glycoprotein precursor, and RNA polymerase had the same size as sizes of published sequences of ANDV (*10**–**12*). The highest degree of identity for the 4 proteins was for virus from NRC2. We compared predicted amino acid sequences with all available complete sequences of ANDV variants circulating in Argentina (South, Lech, BsAs, and Orán). Virus from P1, P2, P3, and NRC2 had 2 identical amino acid differences (T641I and T938A) in the predicted glycoprotein precursor. These differences were not found in any other sequences analyzed. Minor amino acid changes could have major effects on virus properties. Whether an amino acid substitution in viruses from HPS case-patients could determine the person-to-person transmission mechanism should be addressed by comparative analysis of higher numbers of complete virus sequences and specific studies on ANDV transmissibility.

Future studies are needed to obtain additional complete viral sequences from rodent populations co-circulating in the same geographic area. This information will enable definitive differentiation of person-to-person transmission from co-exposures in patients with similar activities of risk in disease-endemic regions, which was the case for P1 and P2, both of whom reported collecting firewood in a forested area.

## Conclusions

We characterized the complete genome of an ANDV strain involved in a person-to-person transmission chain by using target-specific whole-genome sequencing. Our study contributed useful data for clarifying properties involved in the unusual transmissibility of ANDV. These data are crucial for optimal management of HPS case-patients and control of future outbreaks of this lethal disease.
